# Impact of peripheral skin cooling on neuroendocrine leukocytic and hematological reactions during Hypergravity

**DOI:** 10.1038/s41526-025-00486-9

**Published:** 2025-07-02

**Authors:** Michael Nordine, Niklas Kagelmann, Jan Kloka, Hanns-Christian Gunga, Viktor Heinz, Niklas Pilz, Oliver Opatz, Tomas L. Bothe

**Affiliations:** 1 Department of Anaesthesiology, Intensive Care Medicine and Pain Therapy, Goethe University Frankfurt, University Hospital Frankfurt, Theodor-Stern Kai 7, 60590 Frankfurt, Germany; 2 Institute of Physiology, Center for Space Medicine and Extreme Environments Berlin, Charité –Universitätsmedizin Berlin, Berlin, Germany

**Keywords:** Physiology, Preclinical research

## Abstract

Optimal neuroendocrine responses are essential during hypergravity (+Gz) exposure. Peripheral skin cooling (PSC) may enhance neuroendocrine function, potentially improving +Gz resiliency and influencing leukocyte and hematologic factors. This study investigated whether PSC augments the cumulative +Gz stress index (CGSI) and shifts it toward noradrenergic dependency. Eighteen men underwent a graded +Gz profile in a crossover design, with PSC applied using Arctic Sun cooling pads. Neuroendocrine and blood profiles were assessed pre- and post-+Gz. CGSI did not differ between groups, but serum osmolality increased only in PSC (*p* = 0.03). In PSC, CGSI correlated with norepinephrine (*p* < 0.01, *r* = 0.71) and other markers, suggesting enhanced norepinephrine responsiveness despite similar serum levels. This response may be cardio-protective for space missions and ICU patients. Additionally, baseline serum metanephrine emerged as a potential marker for +Gz resilience, with PSC showing potential leukocytic and hematologic involvement in CGSI.

## Introduction

Exposure to hypergravity (+Gz) is an environmental challenge encountered during human spaceflight, and occurs during launch, orbital re-entry, and aerobraking maneuvers^1^. Crucially, the physiological impact of +Gz are most pronounced during the return to normal or partial gravity following periods in microgravity, where cardiovascular deconditioning—characterized by decreases in blood volume, cardiac systolic function, systemic vascular resistance and baroreflex activity^2^—compromises +Gz resilience^3^.

During launch and re-entry, crews may experience +3Gz^4^ for extended periods. During these critical phases, cerebral and cardiac perfusion may be at risk due to the cranial to caudal blood volume shift^5^. This effect persists until the body has been fully re-adapted to planetary gravity. Future space missions, potentially landing astronauts in challenging environments on other celestial bodies, underscore the need for fast-acting, non-invasive and effective countermeasures, ensuring astronauts safety and operative capacity^6^.

The resilience to +Gz hinges on optimal neuroendocrine functioning, particularly through noradrenergic and adrenergic pathways. In response to +Gz, noradrenaline release from the postsynaptic cleft enhances systemic vascular resistance, inotropic cardiac activity and coronary tone. Additionally, renal vasoconstriction activates the renin-angiotensin system and vasopressin release. The adrenergic response works in tandem with noradrenergic pathways to preserve cerebral perfusion via heart rate increases and facilitating vasodilation in specific organ systems^7^. Dysregulation of these responses can lead to physiological instability, posing a threat to mission success.

Research indicates that reduced +Gz resilience or orthostatic intolerance, often stems from inadequate noradrenaline-mediated vasoconstriction in the lower extremities and splanchnic area^3^. This condition is linked to suboptimal increases in total peripheral resistance and more prevalent after longer International Space Station missions and in female astronauts^8–10^. Orthostatic intolerance can even manifest even after short parabolic flights, and is attributed to an ineffective total peripheral resistance response^11^.

Peripheral Skin Cooling (PSC) emerges as a promising countermeasure to augment total peripheral resistance: PSC dramatically boost noradrenergic activity with minimal impact on adrenergic activity^7^, inducing a blood shift from the periphery to the core. This shift results in elevated mean arterial pressure, cardiac output, stroke volume, and systemic vascular resistance^12^. Notably, PSC also induces significant celiac, mesenteric, and renal vasoconstriction. These shifts are highlighted by changes in the balance between noradrenaline and adrenaline, as well as pro-inflammatory markers^13,14^. PSC has also been effectively used to augment orthostatic stability in tilt-table test^15^ and lower body negative pressure (LBNP) studies^16–21^. These studies showcase the efficacy and potential of PSC in improving orthostatic tolerance and hemodynamics during orthostatic stress conditions. However, PSC and its impact on +Gz resilience, neuroendocrine, leukocytic and hematological parameters remain unclear.

We hypothesized that PSC would enhance the cumulative +Gz stress index (CGSI) and noradrenaline levels during graded +Gz exposures in a Short Arm Human Centrifuge (SAHC) compared to a control group (CTL) undergoing the same +Gz profile without PSC. We also anticipate that the increase in noradrenergic activity induced by PSC will affect leukocytic and hematological factors, shifting the CGSI towards a noradrenergic-dominant response. This study aims to evaluate PSC’s potential as a neuroendocrine and hematologic modulator in +Gz environments, a relatively unexplored area in aerospace medicine.

## Results

### Univariate analysis

**Fig. 1:**
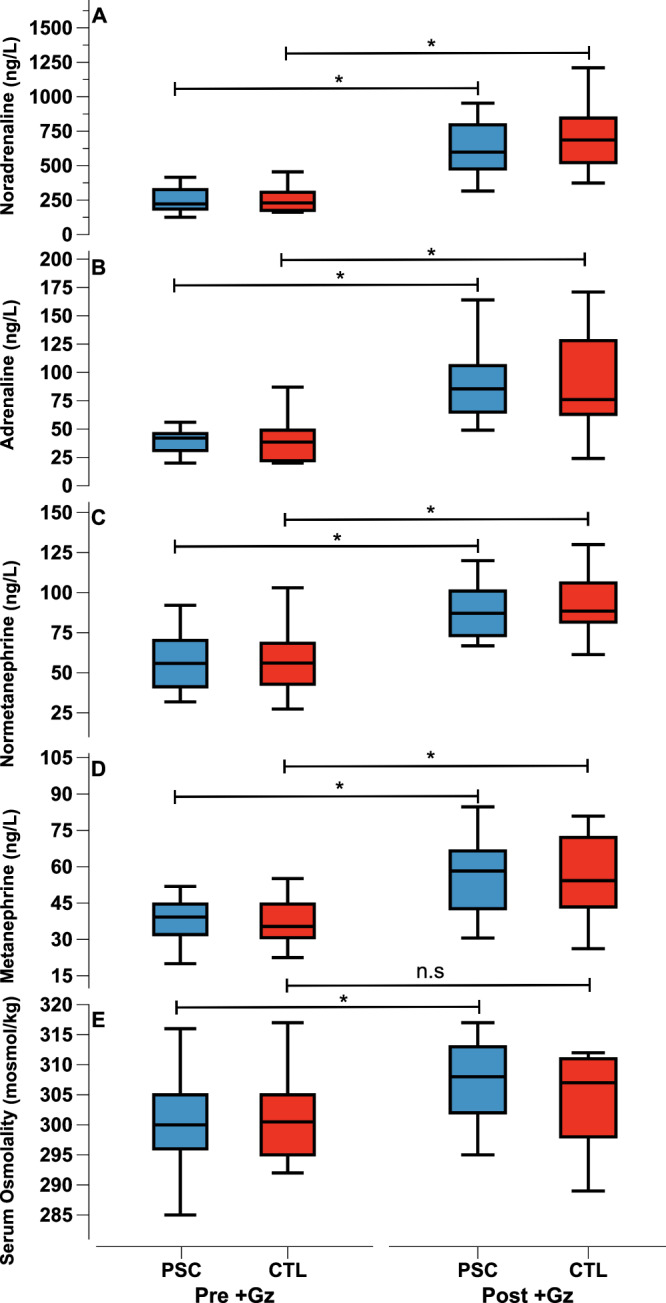
Neuroendocrine parameters. Median values as Tukey style box plots for PSC (Blue) and CTL (red) for Pre +Gz (left) and Post +Gz (Right). From top to bottom: (**A**) Noradrenaline, (**B**) Adrenaline, (**C**) Normetanephrine, (**D**) Metanephrine, and (**E**) Serum Osmolality. Asteriks denote *p* < 0.05 between pre and post +Gz values (paired *t* test).

All 18 participants successfully completed both the PSC and CTL +Gz runs. Consistency in cohort demographics was maintained, as reflected in Table 1, due to the study’s cross-over design. The mean CGSI was comparable between the groups, with values of 1158 ± 327 for PSC and 1116 ± 335 for CTL (*p* = 0.7). Post +Gz, significant increases in noradrenaline, adrenaline, normetanephrine, and metanephrine levels compared to pre+Gz levels were noted in both groups. Notably, post +Gz serum osmolality showed a significant increase compared to pre +Gz only in the PSC group. Further details on these neuroendocrine and hematological changes are provided (Fig. 1)/(Supplementary Tables 1 and 2).

**Table 1:** Anthropometric parameters, entire cohort

Parameter	Mean Value and SD
Age (years)	28 ± 6.4
Height (cm)	180 ± 7.4
Weight (kg)	80 ± 6.9
Body surface area, BSA (m2)	2.01 ± 0.12
Body mass index, BMI (kg/m2)	24.7 ± 1.6
Lean body weight, LBW (kg)	62.7 ± 5.0
Total body volume, TBV (L)	5.34 ± 0.4

**Fig. 2:**
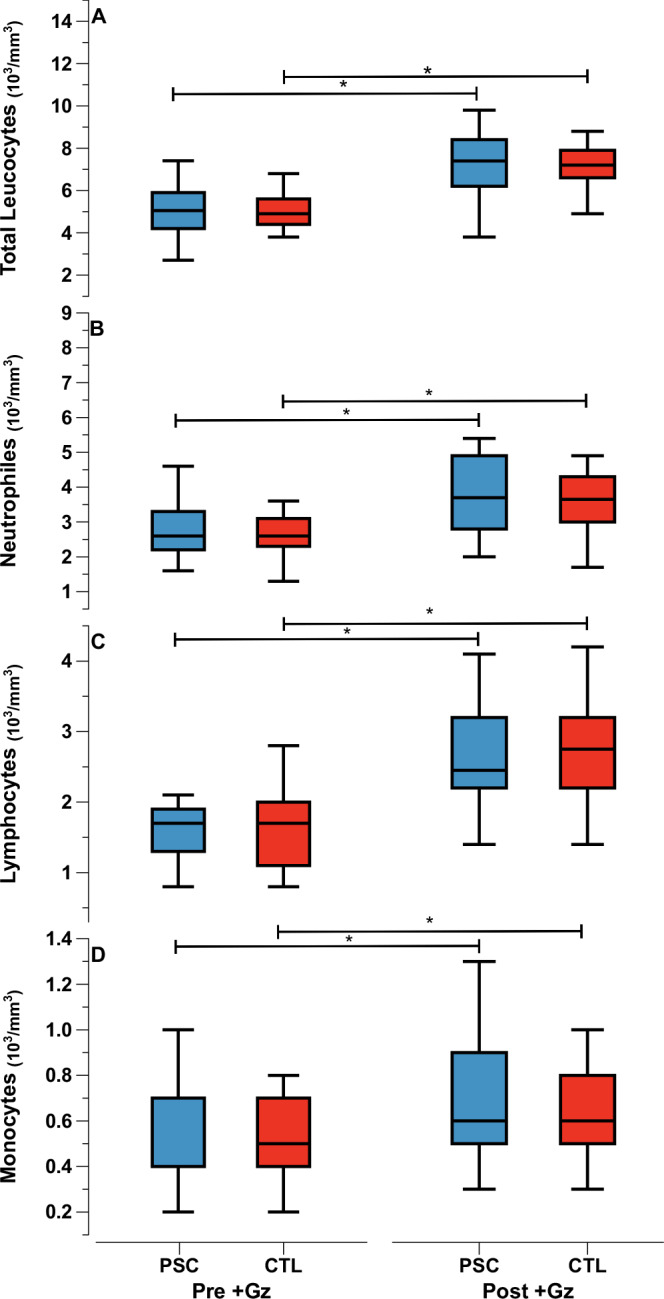
Absolute leukocyte parameters. Median values as Tukey style box plots for PSC (Blue) and CTL (red) for Pre +Gz (left) and Post +Gz (Right). From top to bottom: (**A**) Total Leukocytes, (**B**) Neutrophiles, (**C**) Lymphocytes, and (**D**) Monocytes. Asteriks denote *p* < 0.05 between pre and post +Gz values (paired *t* test).

Leukocyte and hematological responses post +Gz revealed notable patterns. Absolute leukocyte counts demonstrated an equivalent increase compared to pre +Gz values across all parameters in both PSC and CTL groups (Fig. 2).

**Fig. 3:**
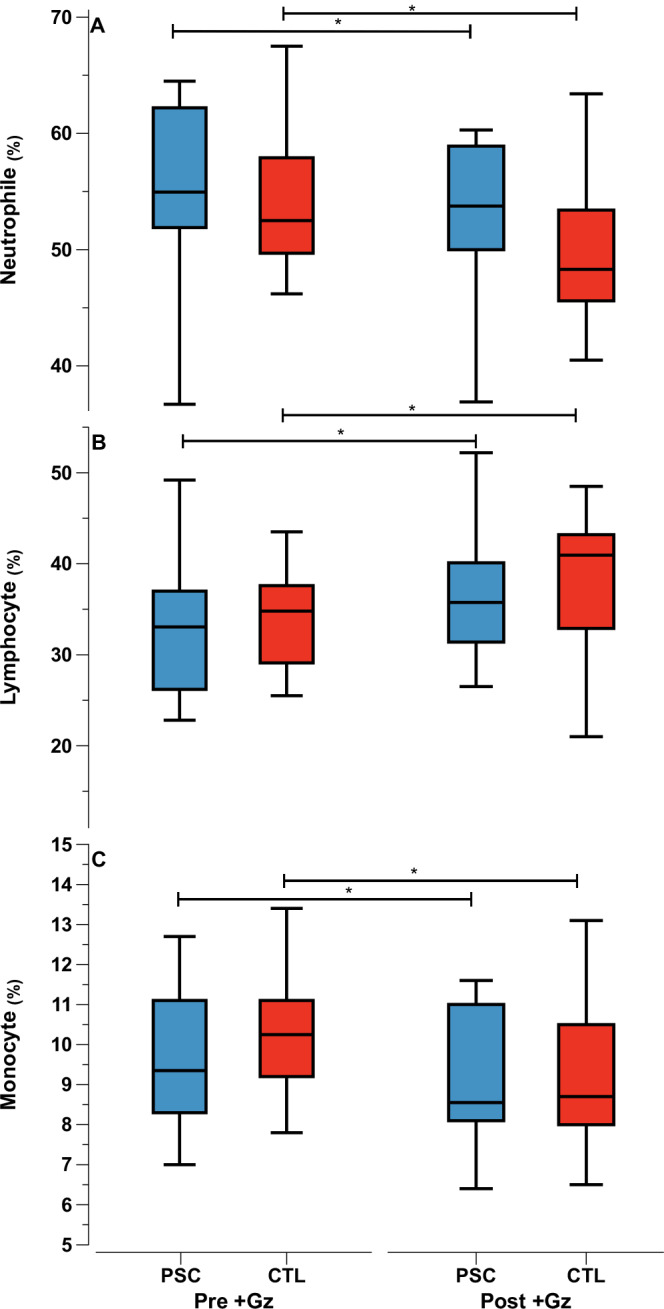
Percentage leukocyte parameters. Median values as Tukey style box plots for PSC (Blue) and CTL (red) for Pre +Gz (left) and Post +Gz (Right). From top to bottom**:** (**A**) Neutrophiles%, (**B**) Lymphocyte%, (**C**) Monocytes%. Asteriks denote *p* < 0.05 between pre and post +Gz values (paired *t* test).

In contrast, the percentage distribution of leukocytes showed a significant shift in both groups compared to pre +Gz levels (Fig. 3).

There was a marked decrease in the percentage of neutrophiles and monocytes, coupled with an increase in lymphocytes. The magnitude of monocyte increase was significantly greater in the PSC group, suggesting a differential impact of PSC on specific leukocyte subtypes (Supplementary Table 2).

**Fig. 4:**
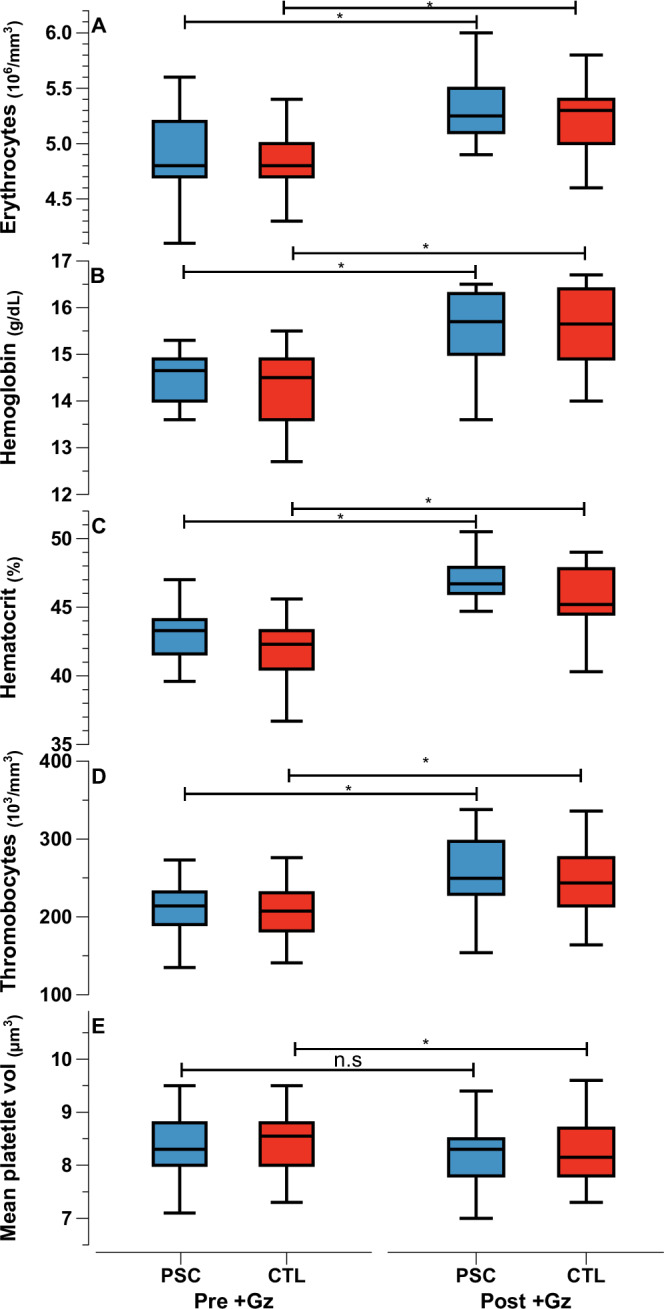
Hematological parameters. Median values as Tukey style box plots for PSC (Blue) and CTL (red) for Pre +Gz (left) and Post +Gz (Right). From top to bottom**:** (**A**) Erythrocytes, (**B**) Hemoglobin, (**C**) Hematocrit, (**D**) Thrombocytes, and (**E**) Mean Platelet Volume. Asteriks denote *p* < 0.05 between pre and post +Gz values (paired *t* test).

Hematological parameters, increased from pre +Gz levels in both groups in general, reflecting a consistent hematological response to +Gz (Fig. 4). However, an exception was observed in the mean platelet volume, which increased significantly only in the CTL group.

### Correlation analysis: CGSI and neuroendocrine/leukocytic/hematological factors

**Fig. 5:**
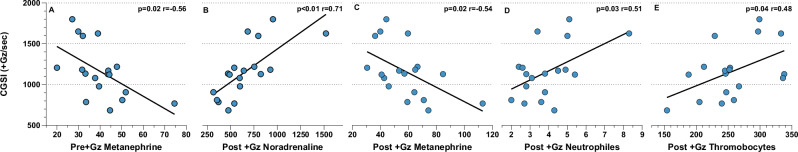
CSGI correlations for PSC. Correlation analysis between (**A**) Pre + Gz Metanephrine, (**B**) Post + Gz Noradrenaline, (**C**) Post + Gz Metanephrine, (**D**) Post + Gz Neutrophiles, and (**E**) Post + Gz Thromobocytes with CGSI for PSC. Denoted are *p* and *r* values (Pearson’s Correlation analysis).

**Fig. 6:**
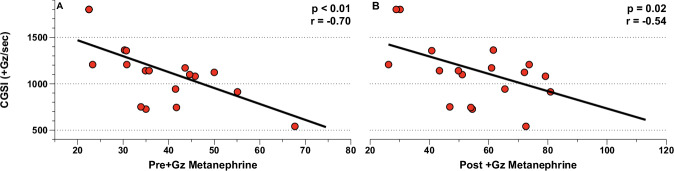
CSGI correlations for CTL. Correlation analysis between (**A**) Pre + Gz Metanephrine, (**B**) Post + Gz Metanehprine with CGSI for CTL. Denoted are *p* and *r* values (Pearson’s correlation analysis).

Pearson’s correlation analysis provided distinct insights for the PSC and CTL groups. In the PSC group, CGSI showed significant correlations with several parameters: pre +Gz metanephrine and post +Gz noradrenaline, metanephrine, neutrophiles, and thrombocytes. This suggests a multifaceted influence of PSC on neuroendocrine and hematological responses under +Gz conditions. In contrast, the CTL group’s CGSI correlated solely with pre and post +Gz metanephrine, indicating a more limited neuroendocrine response to +Gz without PSC. Notably, pre +Gz anthropometrics, along with leukocytic and hematological values, did not demonstrate significant correlations with CGSI in either group, underscoring the specificity of the observed correlations(Figs. 5, 6).

### Correlation analysis: neuroendocrine factors and leukocytic/hematological factors

**Fig. 7:**
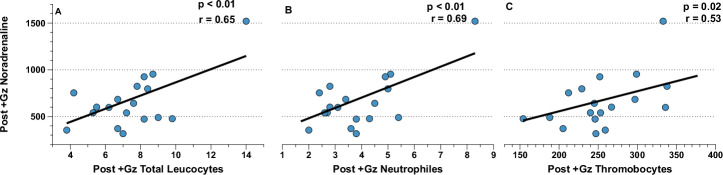
Post + Gz noradrenaline correlations for PSC. Correlation analysis between (**A**) Post + Gz Total Leukocytes, (**B**) Post + Gz Neutrophiles, and (**C**) Post + Gz Thrombocytes with Post + Gz Noradrenaline for PSC. Denoted are *p* and *r* values (Pearson’s correlation analysis).

**Fig. 8:**
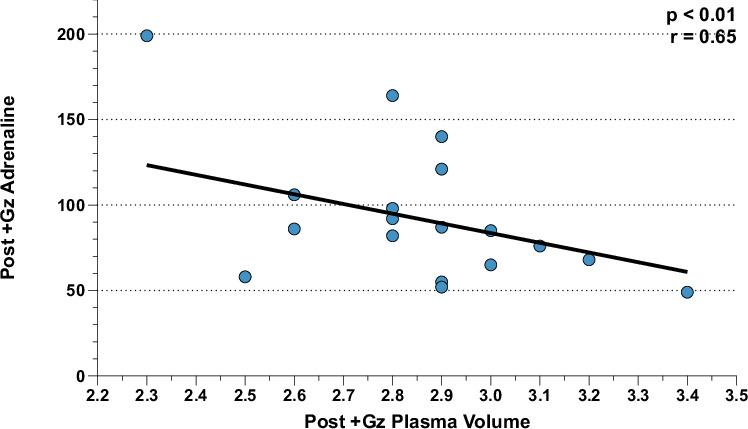
Post + Gz adrenaline correlations for PSC. Correlation analysis between Post +Gz Plasma Volume with Post +Gz Adrenaline for PSC. Denoted are *p* and *r* values (Pearson’s correlation analysis).

**Fig. 9:**
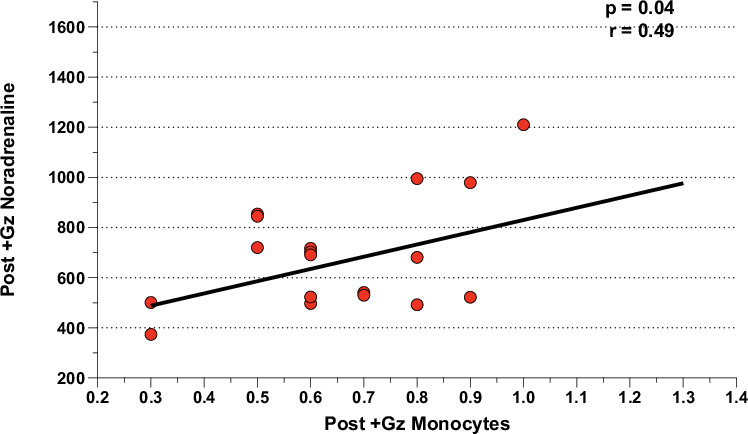
Post + Gz noradrenaline correlations for CTL. Correlation analysis between Post +Gz Noradrenaline with Post +Gz Monocytes for CTL. Denoted are *p* and *r* values (Pearson’s correlation analysis).

Further analysis revealed distinct correlations between neuroendocrine and leukocytic/hematological factors, especially post +Gz. In the PSC group, post +Gz noradrenaline was significantly correlated with total leukocytes, neutrophiles, and thrombocytes, highlighting the interplay between neuroendocrine activation and leukocyte dynamics. Additionally, a notable correlation was observed between post +Gz adrenaline and plasma volume (Figs. 7, 8). In the CTL group, however, the sole significant correlation was found between post +Gz noradrenaline and monocytes (Fig. 9). This highlights a more selective neuroendocrine influence on leukocyte subtypes in the absence of PSC.

## Discussion

This study found that PSC using a bilateral femoral cold-water infusion system did not significantly improve the CGSI or enhance neuroendocrine parameters compared to the CTL during graded +Gz exposure. The neuroendocrine reactions were nearly identical in both groups, except for a notable increase in serum osmolality in the PSC group. Both absolute and percentage counts of leukocytes were consistent across groups, with PSC showing a uniquely greater increase in monocyte count compared to pre +Gz values. This shift indicates a notable change in leukocyte subtype distribution in response to +Gz exposure. Hematological factors were similarly consistent, though CTL exhibited a large increase in mean platelet volume. This differential response might indicate a unique impact of PSC on platelet dynamics under +Gz conditions.

The key finding of this study was the significant correlation between CGSI with noradrenaline in the PSC group, which was not observed in the CTL group. This is further underlined by the significant correlations between neutrophiles and thrombocytes with CGSI in PSC, indicating a synergistic noradrenergic influence. These significant correlations suggest that PSC may augment noradrenaline function as well as optimize its physiological responsiveness during +Gz. Furthermore, this study is the first to demonstrate a correlation between metanephrine levels and CGSI in both groups. The correlations in the PSC group, however, suggest that PSC may shift +Gz resilience towards a noradrenergic pathway rather than relying on a lower adrenergic tone.

These correlations are particularly relevant for enhancing +Gz resilience in human space flight and clinical settings, where PSC’s noradrenaline-mediated cardio-protective properties could be beneficial for longer term missions under medium +Gz conditions. Further, PSC be an important translational finding for supporting the management of hemodynamically unstable ICU patients^22–25^.

Our findings show that CGSI in the PSC group was comparable to the CTL, diverging from LBNP studies that reported enhanced orthostatic stress tolerance with PSC. This difference might stem from the smaller proportion of body surface area covered by PSC in this study, potentially influencing the overall physiological impact on neuroendocrine activity. Furthermore, LBNP and +Gz exert different types of stress: LBNP induces hydrostatic stress, whereas +Gz creates a gravitational gradient along the body, limiting direct comparisons^26,27^.

The graded +Gz profile led to an increase in all neuroendocrine parameters, irrespective of the intervention (PSC or CTL). This uniform response could result from the overwhelming effect of +Gz stress, potentially overshadowing any distinct impacts of PSC. The only directly comparable +Gz study investigating neuroendocrine parameters pre/post +Gz without PSC found a significant increase only in noradrenaline, and a non-significant increase in adrenaline amongst a women and men participants^28^. Earlier research suggested an immediate increase in noradrenaline following PSC application, but this effect diminishes over time^29^. It’s plausible that noradrenaline levels peaked during the initial phases of +Gz in the PSC group. Additionally, studies indicate that during prolonged orthostatic stress, adrenaline becomes the dominant catecholamine, but its vasodilative effect undermines orthostatic stability^30,31^. In contrast, our study observed significant increases in both noradrenaline and adrenaline, which might be attributed to the study’s unique participant profile (male-only) or the intensity of the +Gz profile exceeding +3Gz. It is pre-described that PSC should reduce overall systemic stress, specifically lowering +Gz-induced cortisol activity, which would result in a decrease in adrenaline and metanephrine activity^32,33^. However, this was not observed in the present study. A further consequence of PSC compared to CTL, may be a reduction in overall metabolic and oxidative stress induced by +Gz^34^. A presumptive decline in oxidative stress may have augmented catecholamine responsiveness and immune activity.

Notably, this study also examined plasma normetanephrine and metanephrine, which are inactive metabolites of noradrenaline and adrenaline^35^. These metabolites offer insights into the extraneuronal inactivation of active catecholamines^36^. A previous study involving orthostatic and cold stress reported increases in active catecholamines surpassing those of their inactive counterparts^37^. Our findings partially confirm this, marking the first time these metabolites have been analyzed in relation to +Gz exposure.

A distinct observation within the PSC group was the strong increase in serum osmolality, which has also been observed in previous studies^13,38,39^. This increase in serum osmolality, which can lead to a rapid elevation in vasopressin levels and subsequently blood pressure, might be beneficial for space crews and ICU patients. The initial hemodynamic results suggest that the higher systolic blood pressure trends in PSC could be indicative of vasopressin activation^40^.

There was no marked difference regarding leukocytic and hematological responses between PSC and CTL groups, aside from the magnitude of monocyte change and mean platelet volume. The PSC group indicated a lower change in monocytes. A decrease in mean platelet volume only appeared in CTL. These observations might suggest increased physiological stress in CTL, underlined by the trends in lymphocyte percentages. Consistent with other +Gz studies^41–44^, our study observed leukocytosis and hemoconcentration in both groups. However, the extent of plasma volume reduction was not as pronounced as in other studies^45^. The leukocytosis observed was likely catecholamine-induced due to splenic release and leukocyte mobilization. The use of PSC may have contributed to a reduction in +Gz-associated pro-inflammatory markers (IL-6 and TNF-alpha)^46^ due to an attenuation in overall metabolic and inflammatory stress induced by +Gz^47^.

The correlation analysis revealed strong negative correlations between pre +Gz metanephrine and CGSI in both PSC and CTL groups. Metanephrine was sampled before PSC activation and reflects adrenergic activation independet of PSC’s effects. High metanephrine levels, were associated with lower CGSI. This observation aligns with a previous study suggesting that high baseline catecholamine levels do not predict tolerance to negative pressures up to -50 mmHg LBNP^48^. Conversely, lower renin concentration, often associated with less noradrenaline activation, correlates with reduced orthostatic tolerance^49^. Here, higher baseline levels could equal a lower residual capacity to increased catecholamine or renin levels and therefore a reduced capability to react to increased orthostatic stress. This pattern suggests that metanephrine could be a potential marker for assessing +Gz resilience, warranting further investigation.

A strong correlation between CGSI and noradrenaline in the PSC group points to a unique physiological shift induced by PSC. Despite similar noradrenaline increases in both groups, the correlation suggests a heightened responsiveness or susceptibility to noradrenaline in the PSC group. This implies that PSC might enhance the cardio-protective effects of noradrenaline, potentially beneficial for prolonged use in space missions and in critical care settings. Despite the comparable serum noradrenaline levels in both groups prior to and following +Gz, it is plausible that PSC triggered a localized elevation in cutaneous noradrenaline. This localized elevation could facilitate cutaneous vasoconstriction and possibly enhance the overall sensitivity of the systemic noradrenaline response through the cutaneous neuroendocrine pathway^50^.

Noteworthy correlations were found in the PSC group between CGSI, neutrophiles, and platelets, indicating an interplay between noradrenaline activation and these hematological and immune pathways, which has been previously documented^51–53^. While noradrenaline typically increases neutrophiles, adrenaline usually elevates lymphocytes^42,54^—a correlation not observed in our study. The CTL group showed a distinct pattern, with noradrenaline correlating with monocytes. These differences suggest PSC may amplify neutrophilic responses to +Gz, while CTL links more with monocytic pathways. Such findings resonate with astronaut data, where catecholamine increases post-re-entry correlated with a rise in white blood cells, mainly neutrophiles^4,54^.

Additional correlations in PSC between adrenaline and plasma volume suggest that reductions in plasma volume are crucial for adrenaline release. Cross-talk between neutrophiles, platelets, and plasma volume indicates an interaction between platelet mobilization and volume changes. The correlation between metanephrine and serum osmolality hints at a potential link with the renin-angiotensin-aldosterone system. These results suggest that neuroendocrine activation under PSC leads to a complex cascade of responses involving leukocytic, hematological, and volume homeostasis pathways, underscoring the need for further investigations.

The use of a SAHC in this study to simulate +Gz conditions may not accurately mimic spaceflight associated gravitational transitions, which tend to be longer and less intense. This difference could have influenced the findings, particularly regarding the physiological impacts of PSC on neuroendocrine parameters. The inconsistency in +Gz run durations due to varying maximal tolerance among participants further complicates the uniformity of the experimental conditions.

Subjective decisions by flight physicians to abort +Gz runs introduced variability, especially with a change in physicians midway through the study. This potentially led to different assessments of +Gz tolerance. This subjectivity may have affected the comparability of the PSC and CTL groups.

The water-based PSC system was limited under +Gz conditions due to potential gravitational effects on water flow. Furthermore, limiting PSC application to the bilateral thighs may not have captured the full potential impact of PSC. Covering a larger body surface area could yield more pronounced physiological effects^55^. The use of PSC at 8°C (Arctic Sun minimum temperature) also represented an additional limiting factor. PSC below 8°C may have resulted in a more pronounced elevation in neuroendocrine, leukocytic, and hematological activity due to an augmented thermal response^12^. In contrast, the use of temperatures above 8°C may have exerted minimal to no influence on these parameters. Identifying the most beneficial (both specific to the situation and the person) temperature will be crucial for optimal use in real-world missions.

The study did not include dopamine analysis,s which is also a key neuroendocrine parameter influencing hemodynamic activity during stress. PSC may have had an impact on this pathway, and future studies should include this analysis. Additionally, the methodology of blood sampling only pre and post +Gz, and exclusively from venous blood does not provide a complete picture of the neuroendocrine reactions. This is especially true for neuroendocrine reactions post-PSC activation where reactions may have been more pronounced, or longer-term effects after +Gz exposure, which may have yielded valuable information. Venous blood sampling may also not accurately represent total serum catecholamine levels due to rapid uptake by organ systems and potential local release effects on noradrenaline levels^56^. Although not quantified in this study, post +Gz leukocytic activation and cytokine activation patterns likely increased in both groups^57^, as evidenced by the equilateral increase in leukocytic counts. PSC may have had additional impacts on the aforementioned markers, which may explain why the PSC exhibited a significantly lower decrease in monocyte percentage compared to CTL. Thus PSC may inhibit the migration of monocytes out of the circulation, which could be a result of the impact of cold^58^. Further studies should assess leukocytic activation markers and cytokine patterns during +Gz, with and without a PSC.

In conclusion, this study’s findings reveal that using water-based PSC on the bilateral thighs during graded +Gz exposure did not result in a higher CGSI or a more pronounced neuroendocrine response compared to CTL, with an exception for an increase in serum osmolality. This increase may suggest enhanced vasopressin activity under PSC. Additionally, no differences between PSC and CTL in leukocytic and hematological responses were observed.

However, PSC did lead to a noradrenaline-dependent CGSI. This implyies that while average noradrenaline levels were similar in both groups, its physiological effectiveness during +Gz was enhanced under PSC. The observed correlations between CGSI, neutrophiles, and thrombocytes in the PSC group suggest an amplification of immune-based stress responses. These findings indicate that PSC usage during physiological stress may lead to a more cardio-protective response, beneficial for manned space crews. Additionally, PSC might have stabilizing effects on hemodynamics in clinically unstable patients.

Given these results, future research is warranted to further explore PSC’s impacts using different simulation models like SAHC, parabolic flight, or LBNP. Studies should consider employing alternative PSC systems and extending the application to a larger body surface area. Such research is crucial as non-invasive counter-measure systems will play a key role in the success of human spaceflight and planetary colonization.

## Methods

### SAHC + Gz protocol and participant randomization

This study was conducted at the SAHC facililty at the German Aerospace Institute (DLR) in Cologne, Germany. Eighteen healthy, male civilian volunteers were recruited after a comprehensive pre-+Gz medical examination. Written informed consent was obtained prior to beginning the study. The experiment was conducted in accordance with the Declaration of Helsinki and was approved by the Ethics Committee of the Medical Council North Rhine. The participants, secured in the SAHC in a supine position with their heads toward the gantry center. The ambient temperature was maintained at 23°C – 25°C, and continuous communication was facilitated with the aerospace physician via a microphone and three cameras. Run termination criteria included pre-syncope / syncope symptoms, arrhythmia and/or narrowing pulse pressure. Alternatively, participants were able to stop the experiment at any point using an emergency stop button available for immediate use.

A randomized cross-over design was implemented, with participants divided into two groups (A and B). Group A first underwent a +Gz run without PSC, (classified as CTL), followed by a PSC +Gz run. Group B completed the study in the reverse order. Therefore, each participant acted as their own control (CTL) to their active run (PSC). The participants were also not adapted to cold exposure prior to testing. The minimal +Gz washout period was three days. The SAHC +Gz protocol began with a 10-minute baseline phase at +1Gz and a recovery phase. Then it progressed to a step profile acceleration from +1Gz to +4Gz in 3-minute increments. A detailed graphical description of the +Gz protocol has been previously published^26^.

### PSC application

PSC was administered using the Arctic Sun 5000™ (C.R. Bard, Inc., United States), consisting of a main cooling device and bilateral thigh cooling pads with circulating water at 8°C. To minimize bias, non-cooled pads were used in the control group. PSC commenced during the 10-minute rest period and continued throughout the graded +Gz protocol.

### Neuroendocrine, leukocyte, and hematological parameters

Blood samples for neuroendocrine and complete blood count parameters were collected pre and post +Gz. Neuroendocrine analyses included noradrenaline, adrenaline, normetanephrine, and metanephrine in plasma, as well as serum osmolality (measured in ng/L and mosmol/kg, respectively). The complete blood count, including total leukocyte, neutrophile, lymphocyte, monocyte, erythrocyte, and thrombocyte counts among others, was performed by Axon Lab AG (Switzerland). Plasma and red blood cell volumes pre/post +Gz were calculated using the following formulas: total blood volume in mililiters * hematocrit/100 [red blood cell volume] and total blood volume in mililters * (1-hematocrit/100) [plasma volume]. Total blood volume was calculated according to Nadler^59^.

### Statistical analysis

Within-group comparisons between pre- and post-Gz values were conducted using two-tailed paired *t* tests, while between-group comparisons (PSC vs. CTL) were performed using independent two-tailed *t* tests on absolute and percentage changes from pre-Gz values. A Pearson´s correlation analysis between CGSI and all neuroendocrine, leukocytic and hematological paramaters was performed to assess the relationships of pre and post +Gz for both groups independently. Additionally post +Gz noradrenaline and adrenaline levels were correlated to leukocyte and hematological counts in both PSC and control groups using Pearson’s correlation analysis. Alpha error was set at *p* < 0.05. Statistical analyses were conducted using JASP Version 0.17.2.1 (JASP Team, 2023), with values reported as mean ± SD. Correlation values included *p* and *t* values. Graphical data representation was created using DataGraph Version 5.11 (Visual Data Tools, Inc.).

## Supplementary information

Supplementary Table 1 and Table 2

## Data Availability

The datasets used and/or analyzed during the current study are available from the corresponding author on reasonable request.
