# Supplementary material for: Impact of peripheral skin cooling on neuroendocrine leukocytic and hematological reactions during Hypergravity
Source: NPJ Microgravity. 2025 Jul 2;11:30. doi: 10.1038/s41526-025-00486-9 (PMC12223314; doi:10.1038/s41526-025-00486-9)
Supplement: Supplementary file 1 — Supplementary Table 1 and Table 2 [file 41526_2025_486_MOESM1_ESM.docx]

# **Supplementary Material**

| Parameter | PSC Pre +Gz | PSC post +Gz | %_Change PSC | CTL Pre +Gz | CTL post +Gz | %_Change CTL |
| --- | --- | --- | --- | --- | --- | --- |
| Noradrenaline | 253±21.4 | 658 ±67.2* | 171 ±22.5 | 262 ±24.6 | 687 ±51.6* | 179 ±18.4 |
| Adrenaline | 46 ±6.1 | 94 ±9.5* | 130 ±23.6 | 45 ±6.6 | 88 ±9.7* | 136 ±35.0 |
| Normetanephrine | 60 ±5.5 | 94 ±6.7* | 64 ±7.1 | 57 ±4.6 | 97 ±6.4* | 79 ±9.0 |
| Metanephrine | 40 ±2.8 | 58 ±4.7* | 46 ±7.1 | 38 ±2.8 | 55 ±4.0* | 46 ±7.7 |
| Serum Osmololaity | 301 ±2.0 | 307 ±1.5* | 2.3 ±1.0 | 301 ±1.6 | 304 ±1.8 | 1.0 ±0.8 |
| Plasma Volume | 3.1 ±0.07 | 2.9 ±1.5* | -6.7 ±0.5 | 3.1 ±0.07 | 2.9 ±0.06* | -6.6 ±0.4 |
| RBC Volume | 2.3 ±0.06 | 2.5 ±0.07* | 7.9 ±0.8 | 2.2 ±0.05 | 2.5 ±0.06 | 9.4 ±0.8 |

**Supplementary Table 1: Neuroendocrine parameters for PSC and CTL**. Dispalyed are Pre/Post +Gz Neuroendocrine parameters for PSC and CTL, along with percentage change between pre/post +Gz. Asteriks denotes significant change from pre +Gz levels.

| Parameter | PSC Pre +Gz | PSC post +Gz | %_Change PSC | CTL Pre +Gz | CTL post +Gz | %_Change CTL |
| --- | --- | --- | --- | --- | --- | --- |
| Total leucocytes | 5.3 ±0.4 | 7.5 ±0.5* | 42 ±4.1 | 5.0 ±0.3 | 7.1 ±0.3* | 43 ±3.4 |
| Neutrophiles | 3.0 ±0.3 | 3.9 ±0.4* | 34 ±2.9 | 2.7 ±0.1 | 3.6 ±0.2* | 33 ±3.2 |
| Lymphocytes | 1.7 ±0.1 | 2.7 ±0.2* | 60 ±6.8 | 1.6 ±0.1 | 2.7 ±0.2* | 70 ±6.6 |
| Monocytes | 0.5 ±0.1 | 0.7 ±0.1* | 41 ±5.4# | 0.5 ±0.04 | 0.7 ±0.1* | 25 ±3.9 |
| Neutrophile% | 55 ±1.7 | 52 ±1.7 * | -4.9 ±1.5 | 54 ±1.5 | 50 ±1.4* | -7.1 ±1.7 |
| Lymphocyte% | 33 ±1.7 | 36 ±1.7* | 11.8 ±2.5 | 33 ±1.6 | 38 ±1.7* | 17.3 ±2.8 |
| Monocyte% | 9.6 ±0.4 | 9.1 ±0.4* | -4.7 ±2.1# | 10.4 ±0.5 | 9.2 ±0.4* | -10.7 ±2.1 |
| Erythrocytes | 4.9 ±0.1 | 5.3 ±0.1* | 8.5 ±0.5 | 4.8 ±0.1 | 5.3 ±0.1* | 8.8 ±0.5 |
| Hemoglobin | 14.4 ±0.2 | 15.5 ±0.2* | 8.3 ±0.5 | 14.3 ±0.2 | 15.6 ±0.2* | 8.9 ±0.5 |
| Hematocrit | 42.6 ±0.6 | 46.4 ±0.6* | 8.8 ±0.5 | 42 ±0.5 | 45.8 ±0.5* | 9.2 ±0.5 |
| Thrombocytes | 217 ±10 | 256 ±12* | 18.2 ±1.9 | 214 ±10.2 | 254 ±13.0* | 18.6 ±1.6 |
| Mean corpuscular hemoglobin | 29.4 ±0.5 | 29.5 ±0.5 | 0.1 ±0.1 | 29.8 ±0.4 | 29.8 ±0.4 | 0.03 ±0.1 |
| Mean corpuscular volume | 87 ±1.0 | 88 ±1.0* | 0.6 ±0.1 | 87.2 ±1.0 | 87.5 ±0.9* | 0.4 ±0.1 |
| Mean corpuscular hemoglobin concentration | 33.7 ±0.3 | 33.5 ±0.3* | -0.5 ±0.1 | 34.2 ±0.3 | 34.1 ±0.3* | -0.4 ±0.1 |
| Red blood cell distribution width% | 12 ±0.2 | 12 ±0.2 | 0.2 ±0.4 | 12.1 ±0.2 | 12.1 ±0.2 | -0.1 ±0.4 |
| Mean platelet volume | 8.4 ±0.2 | 8.3 ±0.2 | -1.4 ±0.7 | 8.4 ±0.2 | 8.2 ±0.2* | -2.0 ±0.7 |

**Supplementary Table 2: Leucocytic and Hematological parameters for PSC and CTL**. Dispalyed are Pre/Post +Gz Leucocytic and Hematological parameters for PSC and CTL, along with percentage change between pre/post +Gz.Asteriks denotes significant change from pre +Gz levels. Hashmark denotes significant difference between groups
